# Prenatal Diagnosis of Atrioventricular Block and QT Interval Prolongation by Fetal Magnetocardiography in a Fetus with Trisomy 18 and* SCN5A* R1193Q Variant

**DOI:** 10.1155/2017/6570465

**Published:** 2017-05-30

**Authors:** Lisheng Lin, Miho Takahashi-Igari, Yoshiaki Kato, Yoshihiro Nozaki, Mana Obata, Hiromi Hamada, Hitoshi Horigome

**Affiliations:** ^1^Department of Child Health, University of Tsukuba, Tsukuba, Japan; ^2^Department of Pediatric Cardiology, Ibaraki Children's Hospital, Mito, Japan; ^3^Department of Obstetrics and Gynecology, University of Tsukuba, Tsukuba, Japan

## Abstract

We report a case of fetal trisomy 18 with* SCN5A* R1193Q variant that presented with sinus bradycardia, 2 : 1 atrioventricular block (AVB), and QT interval prolongation. These complex arrhythmias were diagnosed by fetal magnetocardiography combined with ultrasound findings. Advanced AVB and ventricular arrhythmias were confirmed after birth. Genetic testing of the baby revealed a* SCN5A* R1193Q variant, which we considered could account for the various arrhythmias in this case.

## 1. Introduction

Fetuses with trisomy 18 tend to present bradycardia, but further electrophysiological details have not fully been investigated [1, 2]. In the present case, bradycardia and atrioventricular block (AVB) were observed by fetal echocardiography. Further details such as QT interval prolongation and functional AVB due to prolonged ventricular repolarization were accurately diagnosed by fetal magnetocardiography (fMCG). A* SCN5A* gene variant (R1193Q) was identified after birth, which might have attributed to the complex arrhythmias in this patient.

## 2. Case Presentation

**Figure 1:**
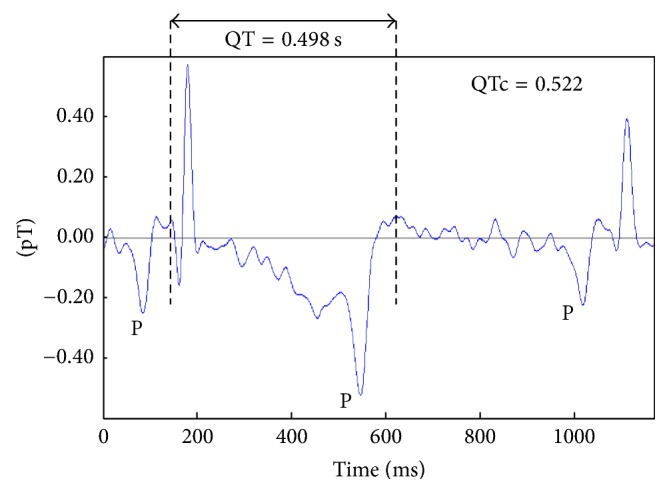
fMCG waveform at 30 weeks' gestation. QT interval was extremely prolonged (normal QT interval of the same gestational week: 0.248 ± 0.316 s [6]). P waves showed the same morphology and polarity. They were observed before the QRS complex and also in the posterior half of the T wave without following the QRS complex, which indicates a functional 2 : 1 AVB related to QT prolongation. pT: piko-Tesla.

The mother was referred to our hospital at 20 weeks of gestation due to fetal bradycardia, fetal growth retardation, oligohydramnios, and overlapping fingers detected on fetal ultrasound. The fetus was suspected to have chromosomal abnormality, specifically trisomy 18. The parents chose not to carry out prenatal genetic diagnosis. Ventricular septal defect (VSD) and bradycardia with AVB were diagnosed by fetal echocardiography. At 28 weeks of gestation, fMCG (MC-6400, Hitachi High-Technologies Corporation) was performed to investigate the details of arrhythmias (Figure 1). Baseline fetal heart rate was around 66 beats per minute (bpm). QT interval was prolonged and 2 : 1 AVB was suspected to be the so-called functional AVB, which develops due to prominently prolonged ventricular repolarization (QT 0.498 s, QTc 0.522). Torsade de pointes or other ventricular arrhythmias were not detected. At the prenatal visit, parents were informed of the possibilities of (1) the need for urgent pacemaker therapy for bradycardia after birth and (2) the occurrence of lethal arrhythmias in late pregnancy or immediately after birth.

**Figure 2:**
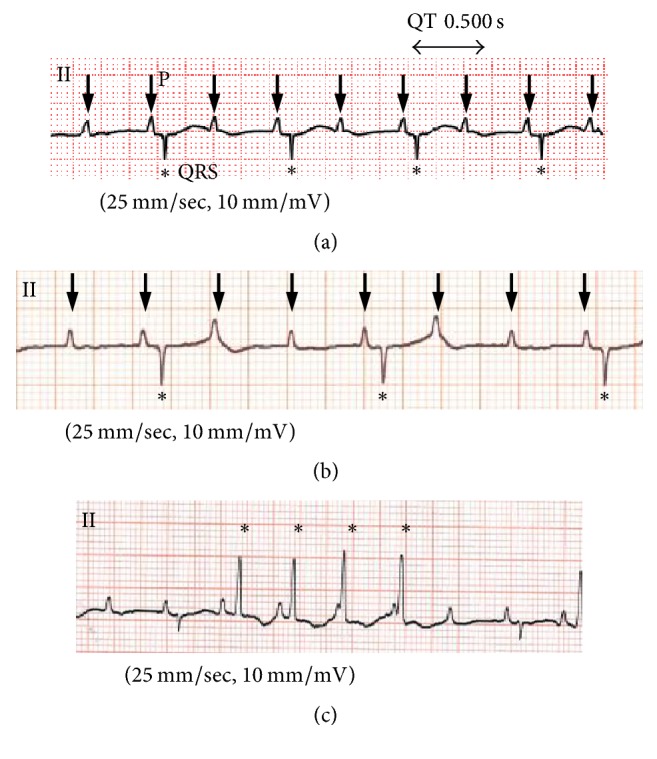
Electrocardiograms recorded after birth (arrows: P wave; asterisk: QRS complex). (a) Severe bradycardia (HR 60–70 bpm), 2 : 1 AVB, and QT interval prolongation. (b) Transient 3 : 1 AVB. (c) Nonsustained ventricular tachycardia.

Fetal hydrops or signs of fetal heart failure were not detected before birth. The baby (male) was born at 37 weeks and 1 day of gestation with a birth weight of 1,211 g. The Apgar scores were 8 at 1 minute and 9 at 5 minutes. Characteristic multiple minor anomalies of trisomy 18 included prominent occiput, micrognathia, and short sternum. Chromosome analysis revealed the karyotype 47, XY, +18 (trisomy 18). A large VSD (inlet type, 4 mm) and patent ductus arteriosus were observed by transthoracic echocardiography. The baby's resting heart rate was 60–70 bpm. A grade of 2 : 1 or higher (transiently, 3 : 1) AVB and QT interval prolongation were recorded on electrocardiogram (ECG) (Figures 2(a) and 2(b)). Nonsustained ventricular tachycardia was also observed (Figure 2(c)). Considering the poor prognosis of trisomy 18, the parents did not consent to aggressive treatments such as pacemaker implantation for bradycardia nor surgical closure of VSD. The patient died at 85 days postnatally, because of progressive heart failure.

Informed consent was obtained from the parents and genetic analysis of long QT candidate genes was performed (Ion PGM™ system, target exon sequencing, and direct sequencing). A* SCN5A* R1193Q variant was identified, which we considered could account for the various arrhythmias observed in this case [3–5].

## 3. Discussion

Trisomy 18 syndrome is usually associated with multiorgan complications and has a poor prognosis. Meanwhile, reports of its electrophysiological details are very few, and whether or not trisomy 18 has arrhythmic substrates is still unknown. To the best of our knowledge, this is the first report to describe complex arrhythmias in trisomy 18. On the other hand,* SCN5A *R1193Q (amino acid substitution of glutamine for arginine), which was identified in this case, is a variant that alters the current of cardiac sodium channel [5]. The R1193Q variant in* SCN5A* has been associated with sudden infant death syndrome (SIDS), long QT syndrome (LQTS) type 3, cardiac conduct defect, and Brugada syndrome [3, 4, 7, 8], and we consider it to be partly responsible for the various arrhythmias that were observed in this case. However, there was no previous report of coexistence of* SCN5A* mutation and trisomy 18. We believe that the coexistent* SCN5A* R1193Q variant in this case was coincidental.

fMCG makes it possible to noninvasively record weak electrical activities of the fetal heart, enabling the measurement of QT intervals of the fetus, and is therefore helpful for differential diagnosis of fetal arrhythmias, including long QT syndrome [9, 10]. In the present case, using fMCG, we made an accurate diagnosis of complex arrhythmias that consisted of sinus bradycardia, AVB, and QT interval prolongation. Based on the detailed information gathered from the fMCG combined with ultrasound findings, we could begin discussions about the treatment strategy earlier within the fetal period.
